# Diagnosis of periprosthetic infection following total hip arthroplasty – evaluation of the diagnostic values of pre- and intraoperative parameters and the associated strategy to preoperatively select patients with a high probability of joint infection

**DOI:** 10.1186/1749-799X-3-31

**Published:** 2008-07-21

**Authors:** Michael Müller, Lars Morawietz, Olaf Hasart, Patrick Strube, Carsten Perka, Stephan Tohtz

**Affiliations:** 1 Charité – University Medicine Berlin, Center of Musculoskeletal Surgery, Berlin, Germany

## Abstract

**Background:**

The correct diagnosis of a prosthetic joint infection (PJI) is crucial for adequate surgical treatment. The detection may be a challenge since presentation and preoperative tests are not always obvious and precise. This prospective study was performed to evaluate a variety of pre- and intraoperative investigations. Furthermore a detailed evaluation of concordance of each preoperative diagnosis was performed, together with a final diagnosis to assess the accuracy of the pre-operative assumption of PJI.

**Methods:**

Between 01/2005 and 02/2007, a prospective analysis was performed in 50 patients, who had a two stage revision because of assumed PJI. Based on clinical presentation, radiography, haematological screening, or early failure, infection was assumed and a joint aspiration was performed. Depending upon these findings, a two stage revision was performed, with intra-operative samples for culture and histological evaluation obtained. Final diagnosis of infection was based upon the interpretation of the clinical presentation and the pre- and intraoperative findings.

**Results:**

In 37 patients a positive diagnosis of PJI could be made definitely. The histopathology yielded the highest accuracy (0.94) in identification of PJI and identified 35 of 37 infections (sensitivity 0.94, specificity 0.94, positive-/negative predictive value 0.97/0.86). Intra-operative cultures revealed sensitivities, specificities, positive-/negative predictive values and accuracy of 0.78, 0.92, 0.96, 0.63 and 0.82. These values for blood screening tests were 0.95, 0.62, 0.88, 0.80, and 0.86 respectively for the level of C-reactive protein, and 0.14, 0.92, 0.83, 0.29 and, 0.34 respectively for the white blood-cell count. The results of aspiration were 0.57, 0.5, 0.78, 0.29, and 0.54.

**Conclusion:**

The detection of PJI is still a challenge in clinical practice. The histopathological evaluation emerges as a highly practical diagnostic tool in detection of PJI. Furthermore, we found a discrepancy between the pre-operative suspicion of PJI and the final post-operative diagnosis, resulting in a slight uncertainty in whether loosening is due to bacterial infection or not. The variation in accuracy of the single tests may influence the detection of PJI. Level of Evidence: Diagnostic Level I.

## Background

The diagnosis of periprosthetic joint infection (PJI) can present a challenge due to the fact that both clinical presentation and preoperative tests are not always obvious and precise. The clinical assessment depends strongly on the symptoms of fever, pain, and fistula. Frequently, persistent pain is the only symptom which is present [1]. Numerous preoperative tests for determination and diagnosis of a failed total hip replacement are available. These tests include haematological screening tests (measurement of the erythrocyte sedimentation rate, and the level of C-reactive protein, white blood-cell count,), aspiration of the hip joint, plain radiography, and radionuclide imaging studies. None of these tests is 100 percent reliable and are subject to a variable spectrum of false negative or false positive results [2]. A misdiagnosis has crucial consequences for the treatment and for the patient. In case of a misdiagnosed periprosthetic infection, revision of the prosthesis without appropriate débridement and antibiosis would keep a failed total hip replacement and risk an early failure. On the other hand a wrong assumption about periprosthetic infection caused by a false positive test, the patient has to undergo surgery which would be highly inadequate as a girdlestone operation (two stage revision) or a cemented revision (one stage revision).

Principal intraoperative tests include the histological evaluation and microbiological cultures of the periprosthetic tissue which have a high validity [3,4]. However the appropriateness of these tests in determining surgical treatment is limited due to the time required to allow for histological preparation and bacteria growth. Hence, final diagnosis of periprosthetic infection can not be made until a couple of days after surgery, when histological and microbiological results are available. Therefore, there is an occasional slight uncertainty whether the right treatment was accomplished.

The aim of this study was to compare pre- and intraoperative diagnostic tests including haematological screening, aspiration, histological evaluation, and microbacterial cultures and to investigate the diagnostic pathway for the detection of periprosthetic joint infection. Additionally, the accuracy of preoperative diagnosis of periprosthetic joint infection was evaluated by comparing preoperative selection with the final diagnosis.

## Methods

### Patients and study design

Between January 2005 and February 2007 a prospective analysis was performed in 50 patients (23 male, 27 female) in the mean age of 69 years (range, 46 – 84) who had a two stage revision because of the suspicion of periprosthetic total hip-joint infection.

Based on the following preoperative parameters, infection of the hip prosthesis was supposed [5,6]:

▪ clinical presentation of infection (Pain, fever, fistula)

▪ plain radiography

▪ haematological screening tests (level of c-reactive protein (>0,5 mg/dl), white blood-cell count (> 12/nl))

▪ early failure within the first five years in connection with clinical or haematological suspicion

When at least one of these parameters was observed, a joint aspiration was performed. Joint aspiration was done according to the standards described in the Guidelines of Hospital Hygiene and Infectious Disease Prevention [7] (Robert-Koch Institut; Berlin), under sterile conditions in an operation theatre, after preparation and draping, using a sterile technique without local anaesthetics and confirming intraarticular placement by radiograph. If no fluid was aspirated, 10 ml of normal saline was injected and reaspirated. If there was a sample of less than 5 ml, only an aerobic bottle was inoculated.

In case of positive aspiration and/or clear clinical or radiographic findings and/or persistence of elevated haematological parameters which could not explained by other conditions, periprosthetic joint infection was diagnosed and a two stage revision has been performed.

### Microbacterial Cultures and Intraoperative Histopathological Evaluation

Intraoperative samples for culture and histological evaluation were obtained from the cup and stem region. Six samples were incubated for 10 days to analyse bacterial growth.

This time period is based on an actual literature review [8-10]. It could be shown that some microorganisms require a minimum incubation time of 8 days, since these microorganisms grow slowly [9,10]. Specimens were inoculated in various culture media (standard eg, blood and chocolate agar, as well as Brain-heart bouillon, Wilkins Chalgren agar, McConkey agar, Sabouraud agar and thioglycolate broth).

A result was considered positive if a minimum of 1 specimen showed growth associated with purulence for virulent organism, such as Staphylococcus aureus, Streptococci species, or a Gram-negative bacterium. For pathogens with low virulence, such as coagulase negative Staphylococci or Propionibacterium species, at least three specimens were required positive (with identical phenotype bacteria profile) for a positive result [11].

The tissue samples for histopathological evaluation were immediately fixed in buffered formalin (4%). Paraffin block sections were obtained (5 μm slice-thickness, slide area 30 × 25 mm) and were stained with haematoxylin and eosin. The slides were postoperatively studied under normal and polarised light microscopy. Evaluation was done using the histopathological classification of the periprosthetic interface membrane according to Krenn and Morawietz [3] (Table 1).

**Table 1:** Definition of the histological types of periprosthetic membranes (Krenn and Morawietz et al.)

**Type**	**Characteristics**
Type I – Periprosthetic membrane of the wear particle induced type	Infiltration of predominantly macrophages and multinuclear giant cells containing PE particles
Type II – Periprosthetic membrane of the infectious type	Activated fibroblasts, proliferation of small blood vessels, oedema, and inflammatory infiltrate of neutrophilic granulocytes
Type III – Periprosthetic membrane of the combined type	Combination of the histomorphological changes described for types I and II
Type IV – Periprosthetic membrane of the indeterminate type (non infected, non wear particle induced)	Connective tissue low in cells and rich in collagen fibres

### Diagnosis of Infection

The final diagnosis of infection was based on the interpretation of the clinical presentation and the preoperative and intraoperative findings rather than on a single test. The final diagnosis of infection was made when the patient met at least one of three criteria: an open wound or sinus in communication with the joint, a systemic infection with pain in the hip and purulent fluid within the joint, or a positive result on at least three tests (Table 2) whereas either histological evaluation or intraoperative cultures had to be positive [4].

**Table 2:** Pre- and intraoperative tests.

▪ Preoperative Tests
- Plain radiography
- Haematological screening tests (level of c-reactive protein (>0,5 mg/dl), white blood-cell count (> 12/nl))
- Aspiration
▪ Intraoperative Tests
- Histopathological Evaluation
- Intraoperative Cultures

At least three of these tests had to be positive whereas either positive histopathological evaluation or positive intraoperative cultures had to be included.

Patients who had neither suspected preoperative signs (as open wound, sinus in communication with the joint, systemic infection with pain in the hip, purulent fluid within the joint) nor had positive results on intraoperative histopathological evaluation or cultures were not held for an infected joint prosthesis.

For the evaluation, the results of every single infection parameter were related to the final diagnosis. On the basis of this relation, sensitivity, specifity, positive and negative predictive values, and accuracy were calculated for each of the tests.

## Results

A two stage revision was performed in 50 patients due to preoperative conspicuousness of clinical presentation and preoperative findings in terms of periprosthetic joint infection. After consideration of the intraoperative test results, final diagnose was made. In 37 patients (74 percent) a diagnosis of periprosthetic joint infection could be made definitely. The time between primary implantation and infection was also measured. In 18 of 37 patients (48.6%) periprosthetic joint infection was diagnosed within the first year after primary implantation, in 6 cases (16,2%) infection occurs between the first and third year and in 13 of 37 patients (35.2%) implant failure was diagnosed after three years. The minimum survival of the prosthesis was one month and the maximum survival was 17 years. In total 31 (62%) of the 50 patients had at least one revision before caused by aseptic/septic loosening, dislocation or wound healing deficits. All hip prostheses were total hip arthroplasties. An analysis of the type and frequency of infecting organisms was conducted and coagulase-negative Staphylococcus (CNS) was found to be the most common (Table 3).

**Table 3:** Type and frequency of infecting organism

**Organism**	**Frequency**	**Multiple existence**
CNS	13	6
Staph. epidermidis	10	4
Staph. capitis	2	1
Staph. haemolyticus	1	1
Staphphylococcus aureus	10	3
Enterococcus faecalis	4	1
Propionibacteria	3	
B streptococcus	2	2
E. coli	2	1
Pseudomonas aeruginosa	2	
MRSA	1	1

Sensitivity, specificity, positive (PPV) and negative (NPV) predictive values, and accuracy for aspiration, intraoperative cultures, histopathology, C-reactive protein and white blood-cell count are shown in table 4. The histopathology yielded the highest accuracy (0.94) in identification of periprosthetic joint infection and correctly identified 35 of 37 infected joint prostheses (Sensitivity 0.94, specificity 0.94).

**Table 4:** Sensitivity, specificity, positive (PPV) and negative (NPV) predictive values, and accuracy for each of the tests are shown.

	***Aspiration***	***Intraoperative*** *** Cultures***	***Histopathology***	***C-reactive*** *** protein***	***White*** ***blood-*** ***cell*** *** count***
**Sensitivity**	0.57	0.78	0.95	0.95	0.14
**Specificity**	0.5	0.92	0.92	0.62	0.92
**PPV**	0.78	0.96	0.97	0.88	0.83
**NPV**	0.29	0.63	0.86	0.80	0.29
**Accuracy**	0.54	0.82	0.94	0.86	0.34

In 13 patients (26%) an infected joint prosthesis could not be confirmed postoperatively (table 5). All 13 patients had clear preoperative findings as pain, early failure, recurrent dislocations or previous revisions caused by aseptic or septic loosening. None of them had an open wound, a fistula or purulent aspiration.

**Table 5:** Pre- and Intraoperative Parameter of patients PJI could not be confirmed postoperatively CNS:

***Patient***	***Preoperative*** *** Findings***	***Aspiration***	***Intraop.*** *** Cultures***	***Histo-*** ***pathology*** *** (Type)***	***C-*** ***reactive*** *** protein mg/dl***	***Prev.*** *** Revision***	**Interpretation**
1	pain, radiogr. loosening	E. coli	-	IV	0.95	2	lowgrade
2	Pain Metal/metal	CNS	-	I	0.6	-	metalosis wear (ME) lowegrade
3	Pain, Radiogr. loosening, chronic bronchitis	CNS	-	I	8.5	1	wear (PE+ME) lowgrade
4	Early failure with in 2 years persistent pain since surgery	Propioni	-	IV	-	-	lowgrade
5	persistent pain	CNS	-	I	-	-	wear (PE) lowgrade
6	pain	CNS	-	I	-	2	wear (PE) lowgrade
7	stem breakage prev. sept. revision	n.a.	CNS	I	-	1	metallosis lowgrade
8	Persistent pain Previous septic revision	-	-	III	-	1	lowgrade
9	Early failure after stem revision by periprosthetic fracture	-	-	IV	-	1	Pseudarthrose
10	Pain Recurrent dislocations Previous septic revision	-	-	I	-	6	wear (PE)
11	Pain recurrent dislocations Metal/metal Failure within 5 years	-	-	I	-	1	metallosis wear (ME)
12	Pain Recurrent dislocation Early failure within 5 years	-	-	I	2.9	4	wear (PE)
13	Pain, radiogr. loosening	-	-	I	1.1	1	wear (PE+ME)

Coagulase negative Staphylococcus; PE: Polyethylen; E. coli: Escherichia coli; ME: metal

(PE: polyethylene, ME: metal, CNS: Coagulase-negative Staphylococcus species, Propioni: Propionibacteria, n.a.: no aspiration)

Six of these patients had a positive joint aspiration whereby 4 showed a growth of CNS, one Propionibacteria and one had E. coli. No pathogen was detected in six cases neither in joint aspiration nor in intraoperative culture. One patient had an intraoperative growth of CNS but showed neither elevated C-reactive protein nor a positive histopathological finding. C-reactive protein was elevated in 5 patients, 3 of them showed a growth in aspiration. In 9 of 13 cases histopathological metal or polyethylene wear particles could be found (type I) and in 3 cases an indifference type (type IV). One patient showed microscopically type III that is containing areas dominated by wear induced antibody reaction and areas with inflammatory reaction caused by granulocytes (lowgrade) but no other suspicious tests were present at this patient. One patient had a positive intraoperative culture and another positive histopathological result, but no other findings. Only three patients had neither elevated C-reactive protein, positive joint aspiration nor positive cultures and positive histological results but at these three patients, an early failure of the joint, persistent pain, previous revisions, recurrent dislocations, were preoperatively noticeable.

## Discussion

The aim of this study was to investigate the accuracy of pre- and intraoperative diagnostic parameters of periprosthetic hip joint infection. Additionally a strategy of preoperative selection of patients with high suspicion of PJI was evaluated and compared with the final postoperative diagnosis.

A limitation of this study is that the study has no control group. This limitation is caused by the fact that there is a lack of a gold-standard definition of periprosthetic joint infection in the current literature. Periprosthetic joint infection is a multimodal process based on different causes and occurs in a variety of clinical presentations. Different diagnostic parameters are published with a broad range of sensitivity and specifity. The Diagnosis of PJI depends on several tests rather than on a single test. An additional problem is that some tests are only postoperatively available such a microbiological cultures or histopathological evaluation. Therefore the preselection of patients with a high suspicion of periprosthetic joint infection is a vast challenge.

In this study the histopathological investigation turns out as a very practical and valid diagnostic tool for intraoperative detection of periprosthetic joint infection with a high sensitivity (0.95) and specificity (0.92). Because of detailed histopathological and polarised characterisation of the periprosthetic interface membrane the clarification whether loosening is due to bacterial infection or not is very precise. A harvesting of tissue samples was possible in all cases. Caused by the study design, tissue samples were only taken in patients with clinical or anamnestic suspicion of infection. A recently published study by Morawietz et al, in which 370 periprosthetic membranes from revision surgery were analyzed, could be shown, that most of the samples (94.9%) were suitable for histological classification [3]. A differentiation of infected and non-infected loosening was well possible. A discrepancy between microbiological and histological findings was found in only 10.7% of the cases.

In 28 of the 37 septic prostheses (75%) the histological and microbiological results were concordant. Similar investigations found a concordance of 89 percent (155/174) [3]. This fact raises the question as to whether microbiological culture or histological examinations are more valid with respect to sensitivity and specificity. Both tests have there individual pitfalls as inappropriate incubation time, previous antimicrobial therapy given to the patient, contamination in terms of cultures or e.g. insufficient preparation of the tissue samples.

To improve the results of histological diagnosis, the surgical pathologist should be provided with additional clinical data, such as the lifetime of the prosthesis, type of fixation, relevant records on clinical pathology, and microbiological findings by the orthopaedic surgeon. This information would help the pathologist interpret results of histopathological samples. Caused by the necessity of tissue sample preparation, an intraoperative statement of the pathologist was not possible in this study. It should be proofed whether the classification system of periprosthetic interface membrane is applicable to frozen section or preoperative biopsy of the neocapsule because a pre- or direct intraoperative test result would be a worthwhile effort. Despite the fact, that the neocapsule is not responsible for loosening, it is generally accepted that the changes in histological appearance are very similar in these different tissue specimens in the same patient caused by interaction of the new joint space with the periprosthetic space [12-14].

Intraoperative cultures are a crucial parameter in diagnosis of PJI and therefore cultures are frequently used as the gold standard to which every other diagnostic parameter is correlated [2,4]. Without correct detection and identification of microorganisms the final diagnosis is ambiguous and adequate antibiotic treatment may not be realized. However, in literature intraoperative cultures have a broad range of sensitivity (range 0.65 to 0.94 (0.78 in this study)) and specificity (range 0.71 to 1.0) (0.92 in this study)) depending on the definition of infection and they are subjected to a variable rate of false positive and negative results [4]. In this study tissue cultures yielded a false result in 8 (16%) of 50 patients (seven false-negative results (14%) and 1 false-positive result (2%)) what is similar to results reported in the literature [12,15,16]. Inadequate incubation time, inappropriate choice of media and antimicrobial therapy, as well as sample contamination from human skin flora are responsible for false-negative or false-positive results. Such problems reduce the level of significance of microbiological culture methods, and have been pointed out in several studies [12,15-18]. It has been reported that, due to the small numbers and low metabolism of bacteria involved in periprosthetic infections generally increase the time needed to resuscitate them [17,18]. Therefore, the growth period should be extended to increase the detection rate of infectious bacteria in excised tissue samples. It could be shown that some microorganisms require a minimum incubation time of 8 days, since these microorganisms grow slowly [10].

Furthermore, it has been emphasized, to improve the hospital culturing of tissue samples, at least eight tissue samples should be taken from different sites in the operative field [19]. Recent studies criticise that traditionally standard diagnostic tests are designed for examining planktonic bacteria and those that are adequate for detecting of sepsis-related pathogens without involvement of foreign material; therefore a significant number of infections of orthopaedic devices may remain undetected [9,17]. The nutrient media and isolation procedures do not provide the requisite conditions for recovering such bacteria in culture.

In this study, preoperative hip aspiration had a low sensitivity (0.57) and specificity (0.5), indicating that indolent joint infection cannot be diagnosed on the basis of aspiration alone. However, it may be the most suitable preoperative tool to provide preoperative information, such as the identity of the infecting organism and it sensitivity to antibiotics [20]. In the literature, preoperative joint aspiration for detecting PJI has a broad range of values of sensitivity varying between 0.11 and 1.00 and specificity varying from 0.78 to 1.00 [2,6,21] certainly depending on the different technique or definition of infection.

Most critical issue in hip aspiration is a high false-positive 6/50 12% and false-negative 14/50 28% rate due to contamination at the time of aspiration or in the microbiology laboratory or false-negative rate due to low concentrations of organisms, delay in transport or inoculating the sample. The inability to aspirate fluid and subsequent washout with saline may contribute to samples with low concentration.

Microorganisms involved in infections of orthopaedic devices are highly adapted on the implant or in the bone-cement interphase, adhering to the environment of the in vivo biofilm, but not planktonic in the synovia and therefore join aspiration may be insufficient [9,17,22].

Our results demonstrate that hip aspiration is only of assistance if there is any conspicuous preoperative sign such as an open wound or sinus in communication with the joint or in case of elevated C-reactive protein which could not be explained by other conditions what is corresponding to the experience of other authors [2].

An elevated C-reactive protein which could not be explained by other conditions highly indicates a PJI especially if there is evidence from clinical or radiographic examination.

In the literature, values of sensitivity and specificity range from 0.61 to 1.0 and from 0.81 to 1.0 [2,4,23,24]. In general, C-reactive protein is a relevant parameter in diagnosing PJI and the elevation of C-reactive protein is a prerequisite to joint aspiration.

Another recommended blood parameter in detection of periprosthetic hip joint infection is the erythrocyte sedimentation rate. In this study we focused our investigation only on the C-reactive protein. Our decision to concentrate on C-reactive protein was reinforced by the fact that the C-reactive protein level increases from normal values to reach maximum values within 24 hours after surgery and then returns to trace amounts in approximately two to three weeks [25,26]. The erythrocyte sedimentation rate may remain elevated for months after an uncomplicated total hip replacement [24]. Therefore, the ability of the C-reactive protein level to return to normal much faster than the erythrocyte sedimentation rate enables it to be a more sensitive indicator of infection, particularly in the early postoperative period.

Definitely postoperative infection could not be confirmed in 13 patients, although there were clear preoperative findings which highly indicated PJI. None of them had an open wound, a fistula or purulent aspiration.

In 6 of these 13 patients the preoperative suspicion of PJI was reinforced by a positive preoperative hip aspiration. But intraoperative microbiological cultures and histopathological results could not confirm the positive results of preoperative aspiration at these patients. It has to assume that aspiration results were false positive.

Also a two stage revision was performed in the remaining 7 patients even though preoperative aspiration was negative. Conspicuous clinical signs like previous septical revision (3 cases), early failure within 5 years (n = 3), and unclear elevated C-reactive protein in connection with persistent hip pain (n = 1) led to septical revision procedure as a precaution not to overlook a creeping infection.

Obviously, there is a difference between the preoperative suspicion of PJI (depending on the clinical presentation and the evaluation of the consultant) and the final postoperative diagnosis that could be made by consideration of all pre- and intraoperative test results. This raises the question of whether the consultant is too prudent in diagnosing PJI or the final definition of PJI is not precise enough or the diagnosis parameters are too insensitive. In consideration of the fact that a miss diagnosed periprosthetic joint infection may have serious consequences for the patient, a minimum of over diagnosed and over treatment is tolerable. Moreover, in case of negative intraoperative test results and absence of fistula, open wound or purulent aspiration, reimplantation would be performed earlier (as soon as test results come up) than it is usual in the standard two stage revision procedure. Negative effects of a two stage revision like muscles atrophy, immobilisation, contractures and leg length differences will be limited to minimum. On the other hand, the relative high number of patients drop out of the diagnosis pattern, could be explained through the definition of PJI. For definition of PJI we tried to include both, clinical presentation and pre/intraoperative test results, similar to the definition of Spangehl et al and Giulieri et al, to obtain a diversified diagnostic pattern [4,27]. But nevertheless, all tests are subjected to a certain rate of false positive and false negative test results, as this and other studies have shown. To obtain highest accuracy in diagnosis of PJI and to improve the detection rate, an exact implementation and interpretation of each single diagnostic test is crucial. Improvements in the technique of joint aspiration, reported by Ali et al, appropriate incubation time of the cultures, the correct choice of media, and stopping previous antimicrobial therapy in advance, are all important points [6]. However, it is recommended that joint aspiration should not be performed, if there is no elevation of C – reactive protein, or clear clinical or radiological signs. Other reasons for elevation of C – reactive protein should be excluded.

Recent investigations have pioneered new techniques for detection of PJI. A notable success in detection of prosthetic hip infection at revision arthroplasty could be achieved by Immunofluorescence Microscopy, Polymerase Chain Reaction (PCR), by analysis of explanted prostheses surfaces and by confocal laser scanning microscopy [18,28,29]. However, it remains to be seen if these new techniques can be established in clinical practice.

## Conclusion

Although periprosthetic joint infection is a well known complication in orthopedics, this study indicates that the detection of PJI is still a challenge in clinical practice. Pre- and intraoperative parameters are subjected to a variety rate of false negative and false positive test results which influences the accuracy of the final diagnosis. In this investigation a discrepancy between the preoperative suspicion of PJI and the final postoperative diagnosis was found that implies in some cases a slight uncertainty in whether loosening is due to bacterial infection or not.

Additionally, one of the most essential facts of this examination is the evidence of the high accuracy of the histopathological classification of the periprosthetic interface membrane in detection of PJI. This classification system proves as a very practical diagnostic tool in detection of periprosthetic joint infection, with both a high sensitivity and specificity.
